# Annotations

**Published:** 1906-06-23

**Authors:** 


### ANNOTATIONS.

### Discovery in Surgery.

Since medicine and surgery concern our bodies, and members of the public in varying degrees take an interest in their physical well-being, the newspaper Press is always on the alert to provide its readers with anything thought to be novel that affects health or disease. While occasionally articles of authoritative value appear, more often than not the information given is untrustworthy. Supposed new facts instead of being new are of old standing; opinions upon debatable or insignificant details are recounted as if well established and all important; and methods of treatment in the tentative stage are given out to the world as if the terrors of some so-far hopeless disease had already disappeared. For the dignity of medicine and surgery this is unfortunate. These sciences need the interest of the public, but that interest is not furthered by the description of wonders where no wonders exist. An illustration of the above remarks is given in such a statement as the following, which has appeared in one of the daily papers: "A revolution has occurred in the surgical treatment of certain conditions of the lung owing to anatomical relationships described by Sir Wm. MacEwen." The anatomical relationships described have long been recognised, at least by many, and their recognition cannot be said to have an important bearing upon the surgical treatment of the morbid conditions of the lung to which the paper refers. The danger of collapse of a lung is rarely present in operations upon abscesses or large tuberculous cavities within it, not on account of the normal adhesive power of the two surfaces of the pleura—which is thought to be a new observation—but because of the presence of fibrous adhesions, and although the opening of such an abscess or cavity usually presents no great difficulty, it rarely materially benefits the patient.

### Infantile Mortality.

It is a somewhat caustic commentary upon our methods that after more than twenty years of compulsory education it should be left to private initiative to instruct the young mothers of our citizens in the elementary duties of motherhood. The ignorance of working-class mothers in the matter of infantile nutrition is phenomenal, a matter the more to be regretted inasmuch as the breast-feeding of babies is becoming progressively less practised. In this fact, of course, lies the root-evil of the whole matter, for dietetical knowledge only becomes necessary (as far as regards the immediate life-and-death interests of the infant) when breast-feeding is abandoned. What is the cause of the decline in breast-feeding? In the case of the rich it is undoubtedly selfishness on the part of the mother, selfishness and lack of maternal instinct. In the case of the poor it is a combination of adverse circumstances of which the chief are the insufficient nourishment of the mothers and the frequency of married women's employment in factories and the like. This aspect of the problem was trenchantly dealt with by Mr. John Burns in his presidential address to the conference on infantile mortality. Married women's labour, he said, manufactured loafers who might be seen buttressing a beershop or French-polishing the outside of a public house; married women's labour was an individual injury, a social mistake, and a commercial blunder. No one who has devoted any time to the study of this subject will hesitate to confirm Mr. Burns's dictum, and it is to be hoped that he will be able, as he desires, to translate his sympathy into action. In the meanwhile there is an obvious need for some organised effort to instruct young mothers in the vital exigencies of infant feeding. Much has been done in individual districts by means of visitors and sanitary inspectors, but much remains to be done. Mr. Burns very truly said that nowhere in the world is young motherhood so isolated as in large cities. A few of these women seek advice at hospitals, but many more act blindly in the light of their own ignorance, or upon the pernicious advice of others who, though as ignorant themselves, yet gather from their riper age a semblance of authority for vicious doctrines.

### Medicine and the Public.

Medicine is not always fortunate in those who take upon themselves the responsibility of providing the profession with a public justification. Excess of zeal, defective judgment, and sometimes a combination of the two, are apt to mark the efforts of the voluntary and unsought champions of many causes, and here the medical profession has suffered with the rest. But there are times of compensation, and such a one it has been pleasant to note within recent weeks. It is not often that a leading daily newspaper presents to its readers within the space of a few days so admirable a presentation of the function of the medical profession as a necessary part of a civilised community as could be read in the letters to the *Times* of Mr. Brodrick, Sir Frederick Treves, and Professor Clifford Allbutt. With the essential material of these letters we are not here concerned. But it is pleasant to recognise a definition of medical opportunity and medical responsibility based upon broad grounds of public polity and including the highest and best forms of professional duty and usefulness. It is in such wise that the profession is truly represented to the public, and it is these ambitions for effective and far-reaching service that secure public respect and confidence. Beside them the noisy arguments of the so-called medical politicians sink into insignificance, and thus reach their true proportions. That day will be a great gain for professional and public interests when it is recognised that the medical practitioner is, as it was once phrased by a great physician, a "naturalist," and that the knowledge which he possesses is gained by the same common-sense methods as those which apply to all other branches of natural history and science. It is because such communications as are above mentioned help to gain this end that, apart from their specific claims to attention, we venture to offer them a word of cordial recognition.

